# Suprascapular Nerve Pathology: A Review of the Literature

**DOI:** 10.2174/1874325001711010140

**Published:** 2017-02-28

**Authors:** Lazaros Kostretzis, Ioannis Theodoroudis, Achilleas Boutsiadis, Nikolaos Papadakis, Pericles Papadopoulos

**Affiliations:** 1 Department of Orthopaedics, Medical School of Aristotle University of Thessaloniki, Thessaloniki, Greece; 2 424 General Military Hospital of Thessaloniki, Efkarpia, Thessaloniki, Greece

**Keywords:** Ligament, Nerve, Neuropathy, Notch, Release, Repair, Review, Rotator cuff, Suprascapular

## Abstract

**Background:**

Suprascapular nerve pathology is a rare diagnosis that is increasingly gaining popularity among the conditions that cause shoulder pain and dysfunction. The suprascapular nerve passes through several osseoligamentous structures and can be compressed in several locations.

**Methods:**

A thorough literature search was performed using online available databases in order to carefully define the pathophysiology and to guide diagnosis and treatment.

**Results:**

Suprascapular neuropathy diagnosis is based on a careful history and a thorough clinical and radiological examination. Although the incidence and prevalence of the condition remain unknown, it is highly diagnosed in specific groups (overhead athletes, patients with a massive rotator cuff tear) probably due to higher interest. The location and the etiology of the compression are those that define the treatment modality.

**Conclusion:**

Suprascapular neuropathy diagnosis is based on a careful history and a thorough clinical and radiological examination. The purpose of this article is to describe the anatomy of the suprascapular nerve, to define the pathophysiology of suprascapular neuropathy and to present methodically the current diagnostic and treatment strategies.

## INTRODUCTION

Suprascapular nerve pathology has recently gained popularity among the causes of shoulder pain and weakness. In 1952 the German neurologist E.Schilf first described a condition of unilateral shoulder paralysis due to the compression of the suprascapular nerve [1]. Kopell and Thompson [2] in 1959 further described the clinical entity of suprascapular nerve pathology. The anatomy of suprascapular nerve and its course through narrow osseoligamentous structures renders it susceptible to compression and traction injuries. Its incidence and prevalence in the population are currently unknown due to the fact that this condition is difficult to diagnose and other pathologies often coexist in the shoulder. Over the past years it has been proposed as an important cause of shoulder pain in overhead athletes [3-7]. Currently reports try to investigate if there is a correlation between suprascapular neuropathy and retracted rotator cuff tears [8-10].

The gold standard study for the diagnosis of the condition are electromyography and nerve conduction studies, whereas computed tomography and magnetic resonance imaging (MRI) are very helpful in visualizing pathological entities in soft and bony tissues respectively. The treatment methods according to the pathology can be surgical and non-surgical.

## ANATOMY

The suprascapular nerve provides motor innervation to the supraspinatus and infraspinatus muscles. Although, it was predominantly recognized as a motor nerve, several anatomical studies have shown that it is responsible for the sensory innervation of the coracohumeral ligament, the coracoclavicular ligament, the subacromial bursa and the posterior shoulder capsule [11, 12]. There are clinical investigations that have demonstrated diminished postoperative pain after a suprascapular nerve bock(SSNB), in patients, subjected to shoulder surgery and support the cadaveric evidence [13-16].

**Fig. (1):**
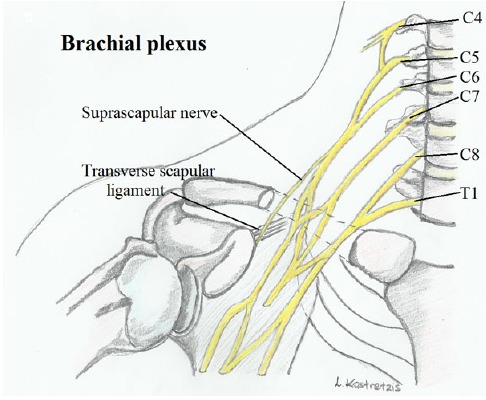
A schematic of the origin of suprascapIular nerve.

The suprascapular nerve emerges from the upper trunk of the brachial plexus and is formed by the venral rami of C5 and C6 roots and sporadically from the C4 root. In a study carried out in 50 Korean cadaveric shoulders by Shin *et al.* [17] the suprascapular nerve was found to originate from the ventral rami of the C5 and C6 in 76.0% of the fifty samples, from C4, C5, and C6 nerves in 18.0%; and C5 nerve in only 6.0%. The nerve courses laterally through the posterior cervical triangle, then travels posterior to the clavicle and finally comes across the superior border of the scapula into the suprascapular notch (Fig. **1**).

**Fig. (2):**
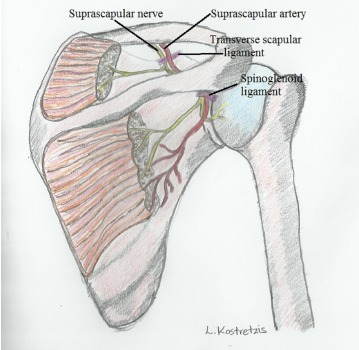
Anatomy of the course of suprascapular nerve.

The nerve runs most frequently through the suprascapular notch underneath the transverse scapular ligament, whereas the suprascapular artery and vein pass over it (Fig. **2**). Several anatomic variations of the suprascapular notch have been recognized and categorized. Rengachary *et al.* [18] in a study of 211 scapulae described six types of suprascapular notch and their incidence as follows: type I depression (8%), type II shallow V-shaped (31%), type III U-shaped (48%), type IV deep V-shaped (3%), type V type III with partial ossification of the ligament (6%), and type VI, complete ossification of the ligament (4%) Fig. (**3**).

The nerve passes through the suprascapular notch 3.0cm medially to the supraglenoid tubercle [19]. As it exits the notch, it crosses the supraspinous fossa providing motor branches for the supraspinatus muscle and receiving sensory branches [19, 20]. The nerve then passes to the spinoglenoid notch under the spinoglenoid ligament, where it supplies motor branches to the infraspinatus muscle [19, 20].

The spinoglenoid notch forms a depression at the posterolateral corner at the base of the scapular spine 1.8 to 2.1 medial to the glenoid rim [19]. According to a cadaveric study by Plancher *et al.* [21] the spinoglenoid ligament was present in all 58 specimens lying 4.8mm over the suprascapular nerve. Another study by the same author proposed that the nerve is being taught in positions of adduction and internal rotation [22].

## PATHOPHYSIOLOGY

There is a wide variety of mechanisms that can cause injury or compression to the suprascapular nerve during its course through the various anatomic landmarks.

The nerve can be compressed or injured in the suprascapular notch and the spinoglenoid notch. As described previously, several anatomic variations of the suprascapular notch can cause stenosis to the space the nerve passes, making it susceptible to compression [19, 23]. In a study of 32 cadaveric shoulders, abnormally oriented fibers of the subscapularis muscle covering the suprascapular notch, anterior coracoscapular ligament and calcified suprascapular ligament were found as possible factors for suprascapular nerve entrapment [24]. Fractures of the suprascapular notch have been associated with lesions of the suprascapular nerve [25] and scar tissue formed following a fracture of the distal clavicle fracture, located proximally to the suprascapular notch, has also been reported to cause nerve entrapment symptoms [26]. Regarding the spinoglenoid notch, a hypertrophied inferior transverse scapular ligament [27] and enlarged spinoglenoid notch veins [28] have been found as predisposing factors of nerve compression in this area and selective atrophy of infraspinatus muscle.

Compression of the nerve can also occur as a result of pathological conditions around the shoulder and at the described positions of vulnerability. Soft and bone tissues masses such as lipomas [29] and intraosseous ganglion cysts [30] have been reported to cause pressure injury to the nerve. Another well described factor for nerve compression in this area are supraglenoid or spinoglenoid cysts (paralabral cysts) whose etiology is closely related to labrar tears and possible glenohumeral instability [29-34]. Turner-Parsonage syndrome also known as idiopathic brachial plexopathy is a viral neuritis that can affect the suprascapular nerve causing shoulder pain and weakness [35].

**Fig. (4):**
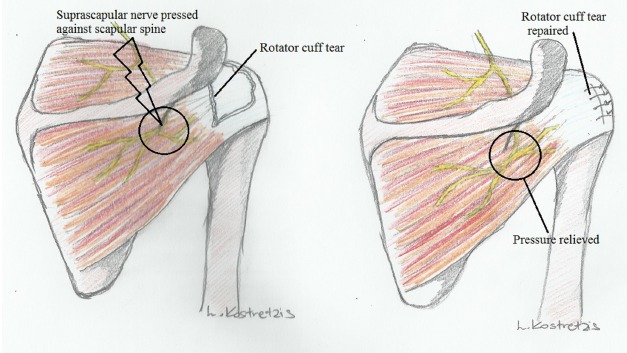
Suprascapular nerve traction following a rotator cuff tear and reversal of pressure after repair of the tear.

Recently, a lot of focus is given toward a possible association between suprascapular neuropathy and retracted rotator cuff tears. Albritton *et al.* in their cadaveric study [10] underlined that medial retraction of the supraspinatus tendon drastically puts the nerve on tension by changing the angle between the nerve and its first motor branch. Clinical studies also imply a relationship between suprascapular neuropathy and retracted rotator cuff tears with recovery of nerve lesions and improvement of function and symptoms when the tears are repaired [36-39] (Fig. **4**).

Nonetheless, there are studies that have shown that there are limits in lateralizing the muscles of supraspinatus and infraspinatus during a repair, as this can set the motor branches of the suprascapular nerve on tension [20, 40]. Warner [20] in his study underlined that supraspinatus can be laterally mobilized as far as 1cm before the motor branches are damaged. Provided the suprascapular nerve is released from the suprascapular notch the supraspinatus muscle can be laterally stretched another 5mm without causing damage to the nerve [20].

Overhead activities can cause dynamic compression to the suprascapular nerve and are well described in volleyball and baseball athletes. In a study the prevalence of suprascapular neuropathy in elite volleyball players was as high as 33% in the dominant hand [40]. There are suggestions that there is association between increased shoulder range of motion and isolated infraspinatus muscle weakness [5]. In the follow up through phase of throwing, the shoulder is in extreme external rotation and abduction and the muscles of supraspinatus and infraspinatus impinge upon the scapular spine compressing the motor branch to the infraspinatus muscle [3, 4]. Another possible explanation for nerve injury in overhead athletes is damage to the axillary or suprascapular artery, causing microemboli to the suprascapular nerve vasa nervorum [41].

Iatrogenic damage to the suprascapular nerve can also occur in cases of distal clavicle excision and open posterior shoulder surgery due to the proximity of the nerve [42, 43].

## EXAMINATION AND DIAGNOSIS

Making a diagnosis of suprascapular nerve pathology is quite difficult from history and clinical examination alone. There are many conditions around the shoulder that can have the same symptoms or can coexist along with suprascapular neuropathy. The clinician should have a high degree of suspicion and a thorough knowledge of the shoulder pathology. A patient with suprascapular nerve pathology will typically present with chronic and dull pain to the superior and the posterolateral aspect of the shoulder, often radiating to the neck or lateral arm. There can be a history of penetrating trauma, previous surgery in the shoulder area and repetitive overhead activity. The patient can also complain about weakness, loss of function and atrophy of the muscles of the shoulder. A high index of suspicion for this diagnosis should exist in overhead athletes such as volleyball, baseball players and swimmers who are at high risk of developing suprascapular neuropathy. Likewise a patient with massive retracted rotator cuff tears can also suffer from this condition.

**Fig. (5):**
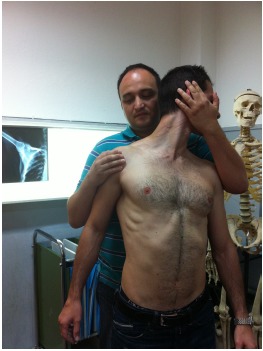
The suprascapular stretch test described by Lafosse.

A thorough and comprehensive clinical examination should follow focusing on both shoulders and neighboring structures such as the cervical spine and the elbow. The patient should be first inspected from the back for scars and for any signs of previous surgery or trauma to the area. Both shoulders are inspected and compared for symmetry, supraspinatus and infraspinatus atrophy or selective infraspinatus atrophy (possible more distal lesion). To rule out cervical pathology a neurological examination is carried out. The supraspinatus fossa and the acromioclavicular joint are palpated for tenderness. The active and passive range of motion is tested and any signs of instability are recorded. A clinical test known as the suprascapular nerve stretch test has been described by Lafosse [44]. The test exacerbates the pain at the posterior aspect of the shoulder that may be caused by constriction of the nerve to the suprascapular notch. The clinician stands behind the patient and gently holds the head of the patient away and in lateral rotation from the affected shoulder while retracting the affected shoulder with his other hand (Fig. **5**). The test is positive when pain is reproduced posteriorly on the shoulder. A very helpful tool to differentiate the diagnosis from other shoulder pathology is the local injection of anaesthetic in the suprascapular or the spinoglenoid notch looking for a relief in shoulder pain.

**Fig. (6):**
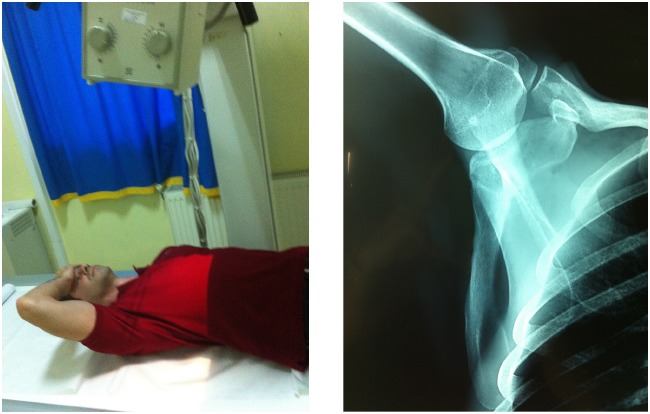
a. Stryker notch view: the patient is supine, placing the palm of his affected hand on top of his head and the beam is tilted 15° cephalad. b. A stryker notch radiograph of the right shoulder.

To continue the evaluation, imaging studies such as plain shoulder radiographs are required. The standard shoulder views: anteroposterior, scapular Y-view and axillary views are examined for shoulder pathology such as fractures of the scapula or the humeral head, signs of rotator cuff arthropathy and bone tumors. A specific view to evaluate conditions in the suprascapular notch is the Stryker notch view and should be included in the radiographic evaluation (Fig. **6**). A detailed computed tomography (1mm slice-thickness) accompanied by a 3D reconstruction could aid drastically in cases of fractures, and anatomic variations offering better visualization of the nerve compression sites.

A Magnetic Resonance Imaging of the shoulder is the best way to visualize the degree of supraspinatus and infraspinatus atrophy [45], to look for soft tissue masses around the shoulder [29], to assess for labrar [46] and rotator cuff pathology and to examine the course of the nerve itself through the bony prominences.

The gold standard study for diagnosis when suprascapular nerve pathology is suspected is electromyography (EMG) and nerve conduction velocity (NCV) studies. In cases of positive clinical and imaging findings or unexplained supraspinatus and infraspinatus muscle atrophy that may suggest suprascapular neuropathy EMG and NCV studies are used to confirm the diagnosis. Findings that are positive for pathology are increased latency, fibrillations and sharp waves that indicate denervation of the supraspinatus and infraspinatus muscles. In an electrodiagnostic testing of patients presenting with weakness, the accuracy of this diagnostic study was as high as 91% [47].

## TREATMENT

By the time the diagnosis of suprascapular neuropathy takes place, there are several treatment options. Non-surgical management is used for the majority of the patients that suffer from an overuse type of neuropathy and no focal mass compression of the nerve. Those with neuropathy due to a rotator cuff lesion and especially with massive, retracted tears usually benefit most from surgical treatment in order to prevent irreversible nerve injury and muscle atrophy. In addition, identifying the region of compression is critical for surgical planning, for which open and arthroscopic techniques may be considered.

### Non-Surgical Treatment

Most physicians recommend an initial nonsurgical approach for most patients with suprascapular neuropathy in the absence of a rotator cuff tear or a space-occupying lesion. The treatment includes, non-steroid anti-inflammatory drugs, activity modification and a comprehensive program of physical therapy, which contains muscle strengthening and increasing range of motion [48-51]. Some patients who had only suprascapular neuropathy profited following a physical therapy program for six to twelve months [50-52].

In a study by Martin *et al.*, fifteen patients with isolated suprascapular nerve pathology were managed non operatively and the follow-up was about four years. Twelve patients had excellent or a good results, while three patients required surgical treatment [48]. On the other hand, in a study by Callahan twenty-three patients with suprascapular neuropathy followed non-operative management without good results and were finally treated surgically [53].

The duration of symptoms and the etiology of entrapment should be considered to determine the length of the initial non-operative treatment. Although, pain reduction and restoration of function are accomplished in most patients [48], once significant atrophy has occurred, muscle bulk and motor strength may be irreversibly lost [54]. The patient that has symptoms for more than 6 months has little opportunity to regain full function if surgery is delayed.

Patients with overuse neuropathy typically do not improve with surgery whereas patients with structural compression of the nerve have been shown to do best after surgical decompression [55]. Therefore, non-operative management is recommended for most patients who suffer from suprascapular neuropathy, particularly in those with an overuse-type etiology [48]. Unfortunately, the patient with a long duration of symptoms, muscle atrophy, entrapment by a mass lesion, and/or associated massive rotator cuff tears has a poorer outcome with a nonsurgical approach [38, 55]. In case of a paralabral ganglion cyst some authors recommend immediate decompression to prevent further nerve injury [54]. Although it is rare, some authors have described spontaneous regression of ganglia [56].

In cases where the nerve is being compressed by a mass or a cyst, conservative treatment may have poorer results. Piatt *et al*. reported that 53% (10/19) of the patients were satisfied following non-operative treatment with pain secondary to a spinoglenoid cyst, compared to 96% (26/27) of patients who were treated operatively [56].

### Surgical Treatment

In case of conservative treatment failure and presence of a reversible, structural cause of entrapment, an operation is warranted, to achieve the decompression of the suprascapular nerve. As mentioned, the most common places of nerve compression are the suprascapular and spinoglenoid notch.

Operative treatment includes decompression of the suprascapular nerve with or without addressing associated shoulder pathology. Some physicians advocate decompression of the suprascapular nerve with simultaneous restoration of rotator cuff tears and labral repair for labral cysts [57-60], while others treat indirectly the pathology by addressing solely the rotator cuff or labral pathology [38, 61, 62]. Isolated suprascapular neuropathy that has not resolved following nonoperative treatment has better results with surgical decompression of the nerve [56, 63, 64].

Mostly, the transverse scapular ligament (TSL) is released and any involved mass is also removed. The TSL can be released through an open incision or arthroscopically. For the open approach a vertical incision 4.5 cm from the posterolateral edge of the acromion can be utilized. Alternatively, a transverse incision anterior and parallel to the scapular spine can be used. The suprascapular notch is approached by elevating the trapezius muscle and retracting the supraspinatus muscle. TSL can now be released [65].

Few complications have been mentioned, most of the patients were free of pain and had recovery of muscle strength, although muscle atrophy did not fully disappear. In a series by Kim *et al.*, 42 patients with nerve injury or entrapment underwent open surgical decompression. Following decompression most of them responded well, had improvement of supraspinatus muscle strength to grade 4 or better and decreased pain was obtained. The study supported that infraspinatus muscle strength recovered less often [66].

Many authors prefer arthroscopic techniques for suprascapular notch decompression [59, 67]. In these procedures, the patient is positioned in the beach-chair position with [59] or without [67] traction. After a diagnostic glenohumeral arthroscopy through a standard posterior portal, a subacromial bursectomy happened when the arthroscope is inserted into the subacromial space . The coracoacromial ligament is exposed medial to the coracoid process after the reposition of the arthroscope into a lateral gate [59] 15 mm medial to the acromioclavicular joint, further medial dissection is performed to the coracoclavicular ligament [68].

**Fig. (7):**
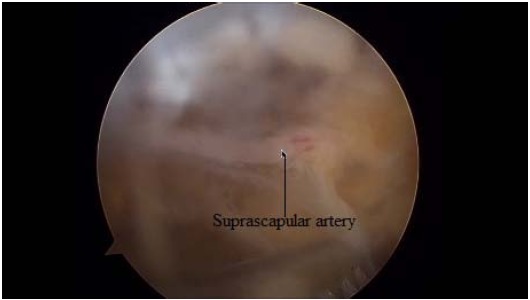
Arthroscopical view of suprascapular artery.

**Fig. (8):**
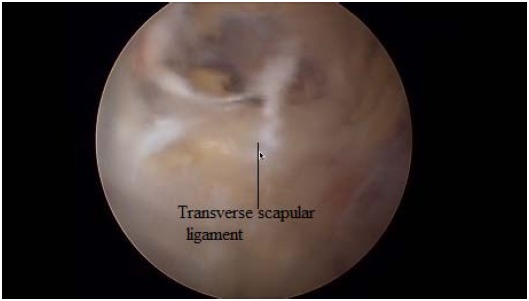
Visualization of Transverse Scapular Ligament.

**Fig. (9):**
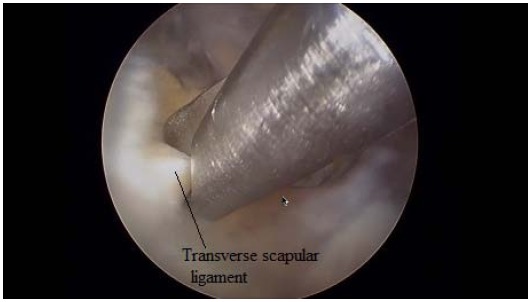
An arthroscopical scissor incised TSL.

**Fig. (10):**
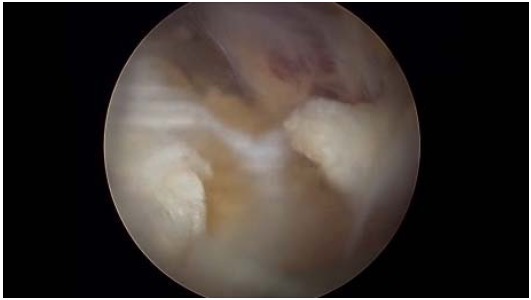
The transverse scapular ligament resected.

**Fig. (11):**
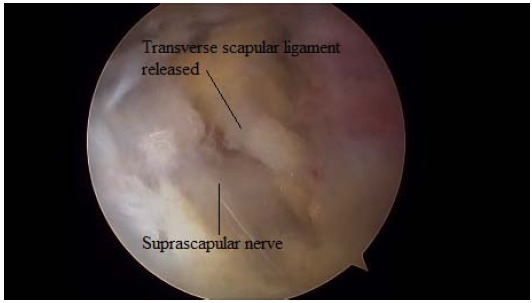
The suprascapular nerve released.

Just medial to these ligaments, the lateral margin of the transverse scapular ligament can be identified. To expose and instrument the suprascapular notch, additional portals are required between the clavicle and the scapular spine (Modified Neviaser portals). Such medial portal is placed approximately 35 mm medial to the acromioclavicular angle, through which a probe and/or elevator are used to bluntly expose TSL and subsequently retract the suprascapular artery (Figs. **7**, **8**), vein and nerve. Another portal is then placed 5 to 10 mm lateral to the previous one, through which arthroscopic scissors are used to perform the actual decompression (Figs. **9**, **10**, **11**). A bony notch resection is needed when ligament release does not adequately mobilize the nerve [59]. An all-arthroscopic technique has been described by Lafosse *et al.* [59]. It utilizes an accessory portal positioned approximately 7 cm medial to the lateral border of the acromion between the clavicle and the scapular spine.

The arthroscopical technique requires high degree of expertise due to the proximity of neurovascular structures. The suprascapular artery must be identified and protected along with the nerve. In a study, in 3% of the patients a subligamentous suprascapular artery was noted [69].

Early results of the arthroscopic approach in ten patients with pathology at the suprascapular notch were evaluated [59]. Seven out of ten patients had a normal EMG postoperatively and the same time, nine patients considered the result as excellent with complete relief of pain.

Regarding the spinoglenoid notch, open decompression is usually achieved through a posterior approach, using an incision 3cm medial to the posterolateral corner of the acromion. After subcutaneous flaps are developed, the deltoid fascia is divided by splitting the muscle, with care taken to avoid dissection more than 5 cm below the acromial border to prevent axillary nerve injury. Retraction of the deltoid facilitates identification of the superior border of the infraspinatus, which is subsequently mobilized inferiorly to reveal the scapular spine. Dissection then occurs above the lateral extent of the spine to release the spinoglenoid ligament and underlying nerve.

Arthroscopic spinoglenoid notch decompressions have been reported, usually in association with the management of paralabral ganglion cysts [70]. Ghodrada *et al.* described a decompression of the suprascapular nerve at the suprascapular notch or spinoglenoid notch through a subacromial approach. Spinoglenoid notch cysts can be visualized through the subacromial space, between the supraspinatus and infraspinatus .

Decompression of a spinoglenoid notch cyst at the base of the scapular spine may be accomplished through a lateral portal whereas a shaver can be inserted through the posterior portal to [71]. On the other hand, Bhatia *et al*. described an intra-articular method for decompression of the suprascapular nerve at the spinoglenoid notch. Decompression is performed with the arthroscope through the posterior portal, while an accessory anterolateral portal is established 2 cm lateral to the anterolateral corner of the acromion and anterior to the anterior aspect of the supraspinatus. Then, a shaver and a switching stick are introduced into the posterior or anterolateral portal and used to probe into the cyst causing an emanation of cloudy fluid for cyst decompression [70].

Westerheide *et al*. treated arthroscopically fourteen patients solely with decompression of the spinoglenoid cyst. At a mean of fifty one months follow up, no complications and no symptomatic recurrences were noted and all had improved strength in external rotation [72].

Lichtenberg *et al*. described eight patients with suprascapular neuropathy due to compression by a cyst in the spinoglenoid notch. Treatment with arthroscopic drainage of the cyst and labral repair (six of the eight) was demonstrated to those who had combined labral lesion (SLAP lesion). In the remaining two patients a capsulotomy was performed to drain the cyst and left open. All patients had pain alleviation and improvement in terms of strength and function [73]. In some cases, a single compression is not obvious, but there are clinical and MRI findings of both supra- and infraspinatus denervation. Under these circumstances, it is not clear if both notches should be decompressed, although there are reports with good results after doing so.

Sandow and Ilic described a treatment with an open posterior and anterior approach so as to access the suprascapular notch and the spinoglenoid notch with excellent results [74]. Soubeyrand *et al.* used an all- arthroscopic technique and they successfully decompressed both notches with a series of arthroscopic portals along the scapular spine [75].

## CONCLUSION

Suprascapular nerve entrapment is an unusual condition causing pain and functional loss in the shoulder. Non-specific symptoms make diagnosis often uncertain. High clinical awareness, imaging studies and electrodiagnostic examination can give further information about the presence of suprascapular neuropathy.

Regarding the treatment of the condition, the location and the etiology of nerve injury are the defining factors. Clinical data are needed to delineate whether decompression of the nerve in cases of rotator cuff tear or compression from a cyst secondary to a labral tear, should routinely be performed.

## Figures and Tables

**Fig. (3):**
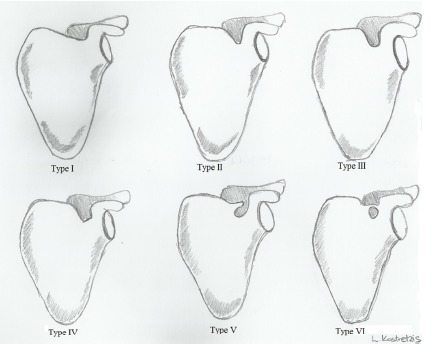
The six types of suprascapular notches.

## LIST OF ABBREVIATIONS

EMG: = Electromyography
MRI: = Magnetic resonance imaging
NCV: = Nerve conduction velocity
SSNB: = Suprascapular nerve bock
TSL: = Transverse scapular ligament
